# Treatment strategies for oesophageal cancer - time-trends and long term outcome data from a large tertiary referral centre

**DOI:** 10.1186/1748-717X-7-60

**Published:** 2012-04-15

**Authors:** Maria C Wolf, Franz Zehentmayr, Michael Schmidt, Dieter Hölzel, Claus Belka

**Affiliations:** 1 Department of Radiation Oncology, LMU University Hospital Munich, Marchioninistraße 15, 81377 München, Germany; 2 Institute for medical Information processing, Biometry and Epidemiology, LMU University Hospital Munich, Marchioninistraße 15, 81377 München, Germany

**Keywords:** Oesophageal cancer, Radio-chemotherapy, Adenocarcinoma, Squamous cell carcinoma

## Abstract

**Background and objectives:**

Treatment options for oesophageal cancer have changed considerably over the last decades with the introduction of multimodal treatment concepts dominating the progress in the field. However, it remains unclear in how far the documented scientific progress influenced and changed the daily routine practice. Since most patients with oesophageal cancer generally suffer from reduced overall health conditions it is uncertain how high the proportion of aggressive treatments is and whether outcomes are improved substantially. In order to gain insight into this we performed a retrospective analysis of patients treated at a larger tertiary referral centre over time course of 25 years.

**Patients and methods:**

Data of all patients diagnosed with squamous cell carcinoma (SCC) and adenocarcinoma (AC) of the oesophagus, treated between 1983 and 2007 in the department of radiation oncology of the LMU, were obtained. The primary endpoint of the data collection was overall survival (calculated from the date of diagnosis until death or last follow up). Changes in basic clinical characteristics, treatment approach and the effect on survival were analysed after dividing the cohort into five subsequent time periods (I-V) with 5 years each. In a second analysis any pattern of change regarding the use of radio(chemo)therapy (R(C)T) with and without surgery was determined.

**Results:**

In total, 503 patients with SCC (78.5%) and AC (18.9%) of the oesophagus were identified. The average age was 60 years (range 35-91 years). 56.5% of the patients were diagnose with advanced UICC stages III-IV. R(C)T was applied to 353 (70.2%) patients; R(C)T+ surgery was performed in 134 (26.6%) patients, 63.8% of all received chemotherapy (platinum-based 5.8%, 5-fluorouracil (5-FU)12.1%, 42.3% 5-FU and mitomycin C (MMC)). The median follow-up period was 4.3 years. The median overall survival was 21.4 months. Over the time, patients were older, the formal tumour stage was more advanced, the incidence of AC was higher and the intensified treatment had a higher prevalence. However there was only a trend for an improved OS over the years with no difference between RCT with or without surgery (p = 0.09). The use of radiation doses over 54 Gy and the addition of chemotherapy (p = 0.002) were associated with improved OS.

**Conclusion:**

Although more complex treatment protocols were introduced into clinical routine, only a minor progress in OS rates was detectable. Main predictors of outcome in this cohort was the addition of chemotherapy. The addition of surgery to radio-chemotherapy may only be of value for very limited patient groups.

## Introduction

Oesophageal cancer is generally associated with poor outcomes. However, over the last decades the treatment algorithms have changed considerably shifting from single mode treatments to complex multimodal approaches [1,2].

Surgery is considered to be the mainstay of treatment especially in earlier stages, however a clear superiority over definitive radio-chemotherapy has not been proven so far [3]. In contrast, for locally advanced stages 5-fluorouracil (5-FU)/cisplatin based radio-chemotherapy (RCT) regimens are the standard of care either in neo-adjuvant or definitive concepts [4-11].

Although several trials and subsequent meta-analysis revealed a benefit for more aggressive approaches - at least in some sub-groups - the benefit in daily routine settings remains poorly defined. Since patients with oesophageal cancer frequently suffer from various comorbidities increasing the aggressiveness of any treatment may only be of value for a very limited subgroup of those patients [6,7,11,12].

The introduction of radio-chemotherapy protocols, regardless of the use of platinum, offer a clear advantage when compared to radiotherapy alone [7,13,14].

The analysis of triple modality approaches leads to a more complex picture: The French FFCD 9102 trial compared radio-chemotherapy plus surgery to radio-chemotherapy alone. Local control and overall survival were almost identical at 2-years with a perioperative mortality of approximately 10% [4]. The authors conclude that there are no clear-cut benefits from the addition of surgery. Data from a similar German trial support these interpretations [9].

Nevertheless, the best outcomes were obtained in those patients with good response after neo-adjuvant RCT and complete resection during subsequent surgery. However, the benefits of improved local control are - at least partially - outweighed by increased surgical morbidity.

A meta-analysis resulted in improved survival rates when the outcomes of RCT followed by surgery were compared with surgery alone [12,15]. However this analysis did not really address the question in how far surgery is needed after good responses to RCT.

The value of most clinical trials on oesophageal cancer is limited to some degree because the clinical status of many of the patients in the 'real-live' setting prohibits the use of aggressive multi-modal protocols and thus the results of the respective trial may not be transferable to most patients.

In order to re-assess the value of different treatment approaches in oesophageal cancer in real life settings we analysed patient characteristics, stages distributions, treatment approaches and outcomes in a cohort of patients treated in one tertiary referral-centre over the last 25 years.

## Materials and methods

### Patients

In a retrospective approach, the following data were systematically retrieved from the original patient files: tumour stage (TNM/UICC version 6), treatment and outcome of all patients with either squamous cell carcinoma (SCC) or adenocarcinoma (AC) of the thoracic oesophagus, excluding AC from cardia and gastric involvement (GEJII+III). All retrospectively collected patient data were compared and crosschecked to data documented prospectively by the population-based Munich Cancer Registry (MCR, documentation started in 1978) for accuracy and completeness to prove the reliability and validity of our data.

Patients were treated between 1983 and 2007 at the department of radiation oncology at the hospital of the Ludwig Maximilian University. For this analysis all patients that presented with oesophageal carcinoma in this department were included, regardless of the form of therapy they received. Overall survival was defined as the survival time from diagnosis to death. Calculations for statistical significance were done only for non-metastatic (M0) patients. A previous published study analysed a subgroup of this collective in order to determine if a definitive RCT with 5 FU and mitomycin C is as effective as the standard protocol with 5 FU/cisplatin [13].

### Statistics

Patient characteristics were compared by the Chi-square test. Survival data were analysed according to Kaplan-Meier (SPSS/WPSS^® ^18.0/19.0). Statistical significance was assessed by univariate and multivariate Cox proportional hazards regression model (p < 0.05) and the log rank test. Patients who were coded as "alive" were censored at the time of last follow-up.

In order to visualize potential time trends, the whole cohort was arbitrarily divided into five treatment periods (five years duration): period I = 1983-1987, II = 1988-1992, III = 1993-1997, IV = 1998-2002, V = 2003-2007.

## Results

### Patients

A total of 503 patients with cancer of the oesophagus were identified. The average number of patients treated per year was 20 (range 6-36). Table 1 shows the patient characteristics of the study population. The median age at diagnosis was 61 years (range 35 to 91 years), 10% were younger than 47 years, 10% older than 77 years at diagnosis.

**Table 1:** Patient characteristics and treatment options, all patients, 1983-2007

Variable	Subgroup	n = 503	%
**Gender**	M	400	79.5
	W	103	20.5
**Age**	< 50	73	14.5
	50-59	158	31.4
	60-69	151	30
	70+	121	24.1
	median y	61	
	range y	35-91	
**Histology**	AC	95	18.9
	SCC	395	78.5
	unknown	13	2.6
**Grading**	G1+2	217	43.2
	G3+4	262	52.1
	unknown	14	2.8
**T**	T1+2	114	22.7
	T3	259	51.5
	T4	90	17.9
	unknown	39	7.8
**N**	N0	203	40.4
	N1	253	50.3
	unknown	47	9.3
**M**	M1	113	22.5
	unknown	28	5.6
**UICC**	I-IIB	190	37.8
	III+IV	281	55.9
	unknown	32	6.4
**Localisation**	cervical	23	4.6
	upper thoracic	114	22.7
	mid thoracic	147	29.2
	lower thoracic	201	40
	unknown	18	3.6
**Therapy**	**prim**.	353	70.2
	RT/RCT	125/227	(35.4/64.3)
	**adjuvant**	51	10.1
	RT/RCT	20/28	(39.2/54.9)
	**neoadj**.	83	16.5
	RT/RCT	17/63	(20.5/75.9)
	unknown	16	3.2
	**RT**	172	34.2
	**RCT**	322	64
	unknown	9	1.8
	**Chemotherapy**	322	100
	5FU+MMC	213	66.1
	5FU+Cisplatin	22	6.8
	5FU	61	18.9
	cisplatin	8	2.5
	unknown	18	5.6
**RT dose in Gy**	≤50	151	30
	> 50-≤54	72	14.3
	≥54- < 60	85	16.9
	≥60	168	33.7
	unknown	17	3.4
**2D/3Dplanning**	2D	289	57.5
	3D	180	35.8
**AL only**		7	1.4

Patients with AC were significantly older with a median age of 65 compared to SCC with a median age of 60 years at diagnosis. 20.8% SCC and 38.9% AC were older than 70 years at diagnosis (p < .0001), they also present a worse grading (p = 0.02) and unfavourable staging with more metastatic disease (p = 0.04). Histology distribution was independent of gender (Table 2).

**Table 2:** Distribution of adenocarcinoma (AC) and squamous cell carcinoma (SCC)

	SCC (n = 395)	AC (n = 95)	p-value
Male	317(80.3)	71(74.7)	0.23
Age			
< 60	195(49.4)	32(33.7)	0.006
60-69	118(29.9)	26(27.4)	
70-	82(20.8)	37(38.9)	< 0.0001
G3+4	192(48.6)	61(64.2)	0.02
T1-2	94(25.5)	17(20.5)	0.33
T3	201(50.9)	51(53.7)	n.s.
T4	73(18.5)	15(15.8)	n.s.
N+	198(50.1)	48(50.5)	n.s.
M1	83(22.0)	28(32.2)	0.04

At diagnosis 311 of all patients (62.5%) were classified as ≥UICC stage IIB, 113 (22.5%) already presented with metastatic disease, 60 (11,9%) were diagnosed with other malignancies such as tumours of the oral cavity, SCLC, bladder cancer etc.

The predominant tumour sites were the mid- and the lower thoracic third with 147 (29.2%) and 201 (40%).

SCC was found in all subsections (26% cervical, 33% mid-oesophagus, 35% distal). AC predominantly in the distal third (65%) of the oesophagus (Table 1).

### Treatment strategy

353 (70.2%) patients were considered to be inoperable because of poor KPS, co-morbidities, locally non-resectable or metastatic disease. 172 (34.2%) received radiotherapy only (RT), 322 (64%) radio-chemotherapy (RCT). Treatment groups were divided in two major categories: RT or RCT as definitive treatment (n = 353, 70.2%) and RT/RCT combined with surgery (n = 134, 26.6%). In the surgery group 51 patients (38%) received adjuvant and 83 (61.9%) neoadjuvant R(C)T. A two-agent chemotherapy (5-FU/MMC) was applied in 235 cases (73%) and either 5FU or cis-platinum in 30.7%. The radiation dose was below 50 Gy in 151 (30%) patients, between 50 and 59 Gy in 157 (31.2%) and in 168 (33.7%) 60 Gy or more. Radiotherapy was applied by a 3 D conformal CT-plan in 180 (35.8%) patients since 1998, a 2 D technique was used before 1998 in 289 (57.5%) cases.

62 patients (12.3%) died during or shortly after treatment i.e. intraoperative (3 patients), after surgery (23 patients (17,2%) from 134 who received surgery), before, during or less than 4 weeks after R(C)T (53 patients (15%) from 353). Considering M0 patients the rate declines to 9.8% for definitive R(C)T but remains the same for the surgery group. In 43 (8.5%) patients treatment was stopped prematurely. The proportion of patients older than 65 who underwent surgery was half of number of patients below 65 (p < .0001). 70% of the patients received chemotherapy in the definitive RT group whereas only 30% in the surgery group (Table 1).

### Time trends

Changes in patient characteristics, therapeutic strategies

The average age at diagnosis increased from 59 y to 65 y. The underlying histology shifted from SCC to AC in our cohort with a significant rise in the prevalence of AC from 16.1% to 27.1% (p = 0.04). In parallel a shift toward more malignant and more advanced tumour stages was observed (grading (p = 0.003), T-stage (p = 0.003), N-stage (p < 0.0001) and - consecutively - UICC stages III and IV (p < .0001) (Table 2). The use of concomitant radio-chemotherapy protocols increased continuously over all time periods, from 37.8% in period I to a proportion of 86.1% in period V (p < .0001) with a two agent approach being used most frequently.

Definitive treatment setting increased from 65.7% in period I to 71% and 78.8% in period III and V respectively. The application of a definitive RCT increased extremely after 1993 with a significant difference comparing time before and after 1998 (p < .0001). A slight decrease in R(C)T combined with surgery can be observed (n.s.).

Higher radiation doses (54 to 60 Gy) were applied significantly more often after 1998.

In order to further validate the results, our own data were compared with the complete data set documented in the MCR. In general, MCR covers the region of central Bavaria, however, these data do not contain detailed radiation data and are restricted to a key set of base line data including histology, stage, general treatment approach and outcome. Thus only parts of the results can be validated using the MRC data. Nevertheless, in the analysis of their own data the MRC reveals a similar gender-, age- and tumour stage- distribution over the time (Table 3).

**Table 3:** Timetrends, all patients distributed in the five equal time periods (5 y each) and compared to available MCR data

Time period		I: 1983-1987	II: 1988-1992	III: 1993-1997	IV: 1998-2002	V: 2003-2007
		n = 143(%)	n = 98(%)	n = 69(%)	n = 108(%)	n = 85(%)
Gender	M	115(80.4)	76(77.6)	52(75.4)	92(85.2)	65(76.5)
	MCR	(84.0)	(83.2)	(83.0)	(79.0)	(80.4)
Age	median y	59	60	59	60	65
	MCR	58	59	61	65	66
Histology	AC/SCC	23(16.1)/117(81.8)	14(14.3)/81(82.7)	12(17.4)/56(81.2)	23(21.3)/81(75)	23(27.1)/60(70.6)
	MCR	(16.5)/(83.5)	(22.9)/(77.1)	(27.6)/(72.4)		
Grading	G3+4	61(42.7)	48(49)	40(58)	64(59.3)	49(57.6)
T	T3+4	76(53.2)	61(62.2)	55(79.7)	90(83.4)	65(76.6)
N1		53(37.1)	36(36.7)	40(58)	65(60.2)	59(69.4)
M1		27(20.9)	17(19.1)	15(22.1)	29(27.6)	25(29.8)
UICC	I-IIB	68(47.6)	45(45.9)	24(34.8)	32(29.6)	21(24.7)
	MCR	(34.4)	(37.0) 1988-2007			
	III-IV	61(42.7)	43(43.9)	43(62.3)	74(68.5)	63(74.1)
	MCR	(51.1)	(63.1) 1988-2007			
	unknown	14(9.8)	10(10.2)	2(2.9)	2(1.9)	1(1.2)
Therapy	definitive	94(65.7)	66(67.3)	49(71)	77(71.3)	67(78.8)
	RT/RCT	55(58.5)/38(40.4)	37(56.1)/29(43.9)	13(26.5)/36(73.5)	10(13)/67(87)	11(16.4)/56(83.6)
	adjuvant	21(14.7)	4(4.1)	9(13.0)	10(9.3)	7(8.2)
	RT/RCT	14(66.7)/4(19)	1(25)/3(75)	3(33.3)/6(66.7)	2(20)/8(80)	0/7(100)
	neoadjuvant	20(14.0)	24(24.5)	10(14.5)	19(17.6)	10(11.8)
	RT/RCT	7(35)/12(60)	4(16.7)/19(79.2)	2(20)/8(80)	3(15.8)/16(84.2)	1(10)/8(80)
RT, M0-patients		56(56.6)	26(36.1)	13(24.5)	9(11.8)	6(10.3)
RCT, M0-patients		43(43.4)	46(63.9)	40(75.5)	67(88.2)	52(89.7)
Surgery, M0-patients		33(32.7)	22(30.6)	16(30.2)	25(33.8)	16(27.1)
RT dose in Gy, M0-pat.	≤54	45(47.4)	36(50)	23(43.4)	38(51.4)	20(33.9)
	> 54 - < 60	4(4.2)	0	6(11.3)	17(23)	27(45.8)
	60+	46(48.4)	36(50)	24(45.3)	19(25.7)	12(20.3)
2D/3D-planning		142(99.3)/0	98(100)/0	65(94.2)/1(1.4)	7(6.5)/99(91.7)	2(2.4)/82(96.5)
Peri-therapy death		19(13.3)	11(11.2)	9(13.0)	15(13.9)	8(9.5)
Therapy break up due to complications		13(9.1)	4(4.1)	7(10.1)	13(12.0)	6(7.1)
Overall survival all/M0	median	18.9/20.7	20.6/26.1	22.6/27.3	20.7/24.3	20.3/29.7
	1 y	(41.1)/(43.7)	(40.2)/(52.2)	(44.9)/(54.7)	(40.7)/(44.7)	(48.8)/(58.7)
	3 y	(7.1)/(8.7)	(15.1)/(18.3)	(14.5)/(18.9)	(17.6)/(19.7)	(15.9)/(19.4)
	5 y	(4.0)/(5.5)	(8.6)/(8.2)	(10.1)/(13.2)	(9.3)/(11.8)	(9.8)/(15.1)

The shift in treatment strategies in the catchment area of the MRC strongly resembles the in house situation: Decreasing rates of surgery, radiotherapy only, decreasing rates of surgery and adjuvant radiotherapy only, increasing use of RCT and RCT+surgery.

### Overall survival and prognosis

**Figure 1:**
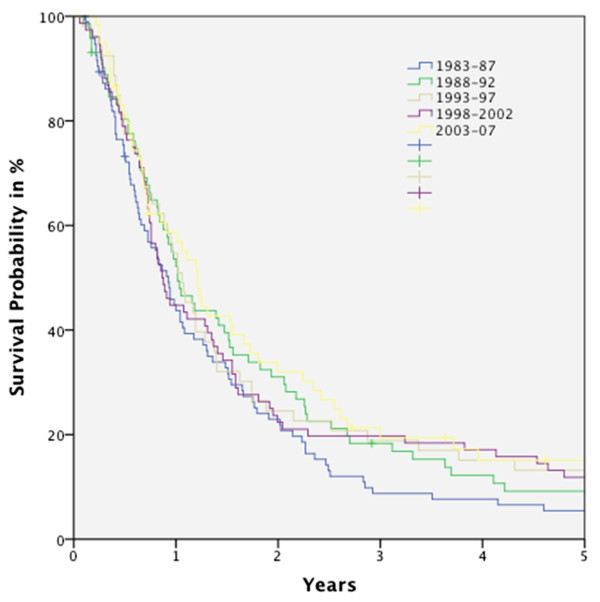
**Kaplan-Meier curve for overall survival (OS) for all M0-patients (n = 362) distributed to the five time-periods**.

**Figure 2:**
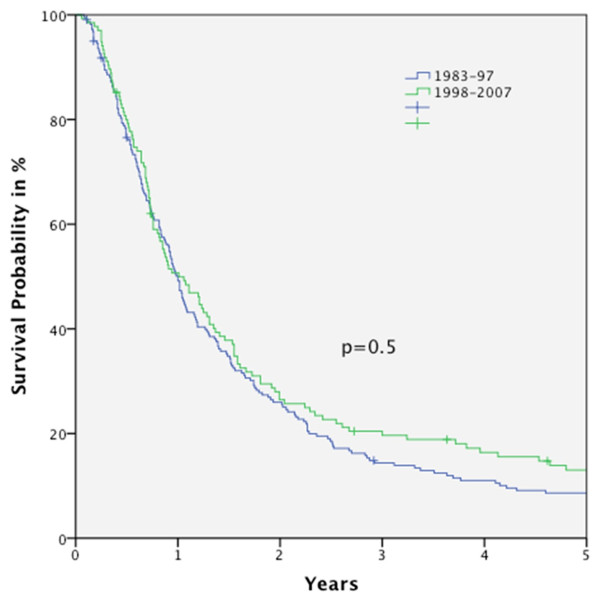
**Kaplan-Meier curve for overall survival (OS) for all M0-patients (n = 362), comparison between OS for patients diagnosed between 1983 and 1997 and between 1998 and 2007**.

**Figure 3:**
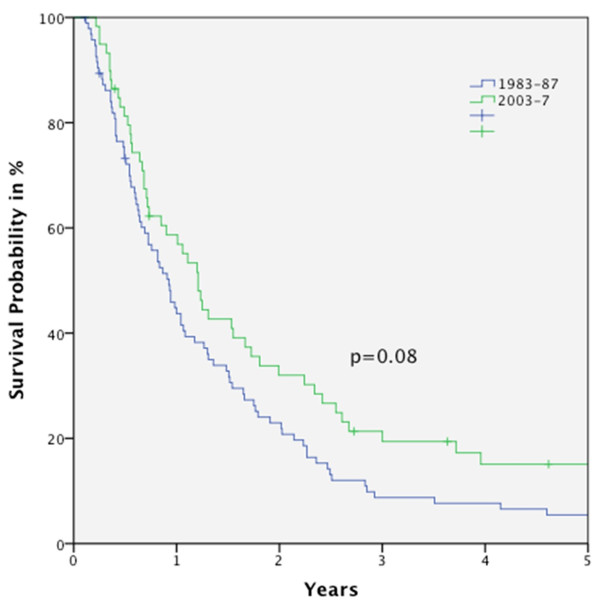
**Kaplan-Meier curve for OS for patients diagnosed in period I (1983-1987) compared to period V (2003-2007), improvement in OS is not significant (p = 0.08)**.

Figure 1 shows the OS curves for the five periods. In general, no statistically significant improvement in OS rate was seen over the time course (Figure 2). Even comparing period I and V, no significant outcome improvement was observed (Figure 3).

A significant improvement of the OS rates was found when outcomes after RCT were compared to those after RT only (p = 0.002), regardless of the chemotherapy protocol used (2 substances vs. single-agent (n. s.)). In parallel, a radiation dose higher than 54 Gy seems to have an influence on survival (p = 0.027), decreasing at doses higher than 60 Gy (p = 0.04) but only RCT remains significant in the multivariate Cox proportional hazard analysis (Table 4)

**Table 4:** Cox Regression Analysis, HR = Hazard Ratio calculated with 95% confidence interval (CI) by Cox-proportional hazard model

		Overall survival	
		Univariate p, HR(95%CI)	Multivariate p, HR(95%CI)
	Comparison		
**Therapy**	prim vs adj+neoadj RCT	0.096, 1.23 (0.97-1.56)	
	RCT vs RT	**0.002**, 0.69(0.54-0.87)	0.02, 0.74(0.58-0.98)
	1 agent vs 2 agents	0.34, 1,04(0.97-1.11)	
**RT dose in Gy**	≥54 Gy vs < 54 Gy	**0.027**, 0.78(0.62-0.97)	0.18, 0.85(0.67-1.089
**2D/3Dplanning**	2D vs 3D	0.71, 0.98(0.76-1.2)	

**Figures 4:**
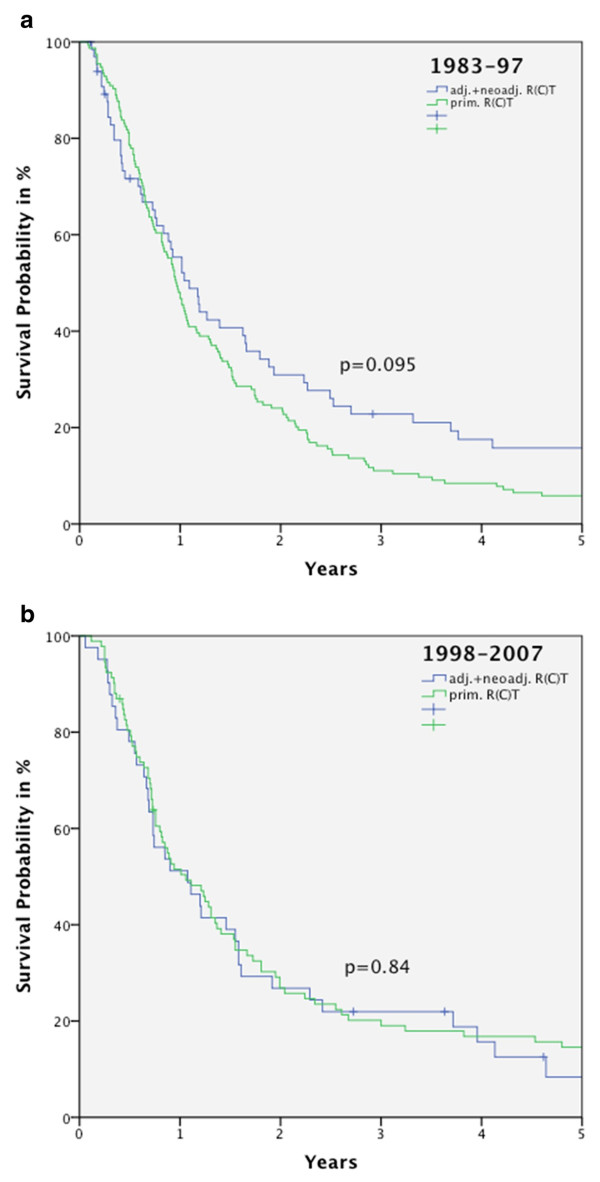
**Kaplan-Meier curves for OS for patients diagnosed between 1983 and 1997 who received R(C)T compared to R(C)T+surgery presenting a better outcome (4a) and presenting no difference in OS in the two therapy groups between 1998 and 2007 (4b)**.

Yet it is highly noteworthy that the superiority in OS for patients in the surgery group seen before 1998 (period I-III) is no longer visible comparing R(C)T versus R(C)T+surgery for patients diagnosed between 1998 and 2007 (period IV+V) and the OS curves converge (Figures 4a, b). When patients received R(C)T an improvement (n.s.) in OS can be observed comparing the periods. There was no improvement when R(C)T+surgery was performed.

## Discussion

Primary aim of this study was to assess changes in basic clinical characteristics, treatment approaches and their impact on survival in a very large cohort of patients followed over a long period of time. Secondly, we analysed patterns of change in the use of radio-(chemo)therapy with or without surgery. For comparison with epidemiological data we included M1 patients but removed them for not compromising the informative value.

In general, there was a clear shift over the observation period regarding fundamental patient characteristics towards higher ages, a higher proportion of adenocarcinoma and towards more advanced tumour stages at diagnosis. Similar tendencies were also visible when analysing the complete epidemiologic data of the Munich Cancer Registry. Stage shift and stage migration is - at least in part - related to an increasing utilisation of improved staging possibilities including Endo-US, MRI and PET-CT [16-20].

The increased rate of adenocarcinoma was already reported by multiple groups world-wide and potentially reflects a completely different pathogenesis [21-27].

However, the shift towards adenocarcinoma is slightly less pronounced when compared to the SEER database (59.2%, n = 16,162; 2004-2008). As opposed to literature [4,28] we detected a worse OS for AC patients than in patients with SCC, possibly because of the negative patient selection in our cohort. The proportion of synchronous malignancies of 11.9% in our cohort is well in line with published data [29,30].

In general, the prognosis of oesophageal cancer is dominated by two competing risks: loco-regional relapse and distant metastases. Surgery as well as radiation as single modality approaches yield 5 y survival rates below 10% [31]. With both approaches local control rates are far from being optimal. For this reason surgery, radiotherapy and chemotherapy were combined in a multimodality approach. As expected, in our cohort therapy options shifted towards multimodality. However, only the combination of radiotherapy with chemotherapy seems to impact on overall survival. This has already been shown in the framework of prospective randomized trials [8,32,33] and also for patients treated along the given trial protocols but formally outside of the randomized trial [7]. However, no such data have been shown for a non-selected patient cohort. Of special notice in this regard is the fact that most patients in the Munich setting have received mitomycin-C [13] instead of cisplatin which was used in most of the randomized trials [7,8,14,33]. Interestingly, the addition of chemotherapy only increases local control and was never found to reduce the high rate of loco-regional and distant seeding.

However, for the whole study cohort the improvements in the field of radio-chemotherapy were not prominent enough to significantly increase OS rates. Thus, despite considerable changes in the treatment regimens over the past decades results are still moderate and only a slight improvement in OS was seen in this unselected patient-cohort.

When trying to determine the role of surgery using our patient cohort some limitations have to be considered: The most striking bias is the fact that all patients have been selected to receive radiotherapy based on clinical reasons. Thus, the value of surgery can only be estimated for those patients. In our cohort, the value of surgery for overall survival seems to be limited. This is in accordance with the results of randomized trial showing that the addition of surgery to combined radio-chemotherapy does not increase OS [3,4,9]. Despite the fact that the best results regarding survival are achieved in patients with triple modality approaches the impact on a larger cohort is limited. This is related to the fact that the increase in mortality by the addition of surgery counteracts the effects of an increased local control. Thus, no clear contribution of surgery to the outcome is visible in our cohort.

Importantly, radiation dose was related to OS-rates in this large cohort. In a previous randomized trial [34] no such correlation was documented. However the value of this trial is strongly limited since most of the excess mortality in the higher dose arm occurred early in the treatment course and several protocol violations were documented. Our findings are in accordance with data published by Geh [35] who has provided evidence for clear dose response relationships for oesophageal cancer based on dose response data compiled from multiple trials.

As already pointed out, the use of a non-selected patient cohort for study purposes is associated with certain shortcomings: It is impossible to control for imbalances due to individual and location specific clinical decisions. In addition, time trends are influenced by general scientific progress but also by centre specific variables. Thus, all interpretations need to be very careful, considering selection bias in this retrospective setting for such a long period of time. The patient characteristics in our unselected cohort are comparable with whole epidemiologic data set of the Munich Cancer Registry (MCR) and published trials. Thus the resulting conclusions are substantiated to some degree. Up to now only very few other trials have tried to approach the value of treatment approaches in oesophageal cancer using population-based data. In this regard a US group supported by the National Institutes of Health used the instrument of a survey to collect demographic data on patient and information on surgical approach for oesophageal carcinoma across the whole country [36]. The evaluation showed that there is a substantial heterogeneity in surgery strategies and emphasized the need of controlled trials to determine best practices. Another study from the US queried the SEER database to prove the benefit of neoadjuvant RT on survival for patients undergoing definitive surgery [37]. Also by SEER-query Chang et al. [28] found no difference in survival and response between AC and SCC across any of the major treatment modalities.

An investigation on trends in treatment and factors influencing treatment receipt and survival were sourced from the Irish National Cancer Registry [38] and showed decreased use of surgery, especially in older patients and a considerable difference between the survival observed at population level and in randomised controlled trials.

The MCR and the SEER registry are population-based databases that represent an unselected group of patients without consistently recorded medical or course information. Therefore only statements concerning epidemiology and outcomes in actual clinical practice outside of the controlled setting of research protocols can be obtained. Although course and recurrence information is poor in our data and also in the databases, overall survival is a good surrogate for disease recurrence, because the outcomes with oesophageal cancer are poor and recurrences in this disease are rarely salvaged. Cause of death and mortality data are available and generally corresponds to disease recurrence.

Despite the fact that the results of several large randomized trials are available the general progress in oesophageal cancer is limited. In the future, several crucial aspects are of importance: A key problem is the fact that a considerable number of patients are not suitable for aggressive approaches [39]. Hence, major efforts should be placed on the development of tools for accurate patient selection according to the individual risk situation and estimated prognosis and reducing therapy associated morbidity and mortality [22,25,31,40,41]. In this regard, important contributions may come from similar disorders including head and neck cancer or lung cancer in which the development of modern imaging approaches as well as biological stratification approaches already dominate the research horizon [42,43].

In parallel, the fate of patients with oesophageal cancer is largely influenced by early dissemination, thus it is of key importance to test the value of an integration of targeted drugs with proven activity in either SCC or AC into putative new treatment protocols [44-46]. Similarly, new radiation techniques suitable to reduce lung toxicity or increase target volume conformity [47-50] will have a clear role in optimizing the outcomes in oesophageal cancer.

## Conclusion

Despite an increase in unfavourable tumour stages a slight (but statistically insignificant) improvement of survival for the whole cohort can be observed, which can be mainly attributed to the addition of chemotherapy to radiotherapy. The role of surgery for most of the patients with locally advanced disease is not fully determined. Despite all efforts in the field of multimodal approaches, prognosis of oesophageal cancer is still limited.

## Competing interests

The authors declare that they have no competing interests.

## Authors' contributions

MCW carried out the retrospective data acquisition, performed the statistical analysis and drafted the manuscript, FZ participated in data analysis, manuscript preparation and revision, MS and DH participated in data acquisition, the statistical analysis and interpretation of data, CB provided the idea, contributed to the conception of the trial, drafting and revision of the manuscript. All authors read and approved the final manuscript.
